# Supplementary material for: R-Ras-Akt axis induces endothelial lumenogenesis and regulates the patency of regenerating vasculature
Source: Nat Commun. 2017 Nov 23;8:1720. doi: 10.1038/s41467-017-01865-x (PMC5700916; doi:10.1038/s41467-017-01865-x)
Supplement: Supplementary file 1 — Supplementary Information [file 41467_2017_1865_MOESM1_ESM.pdf]

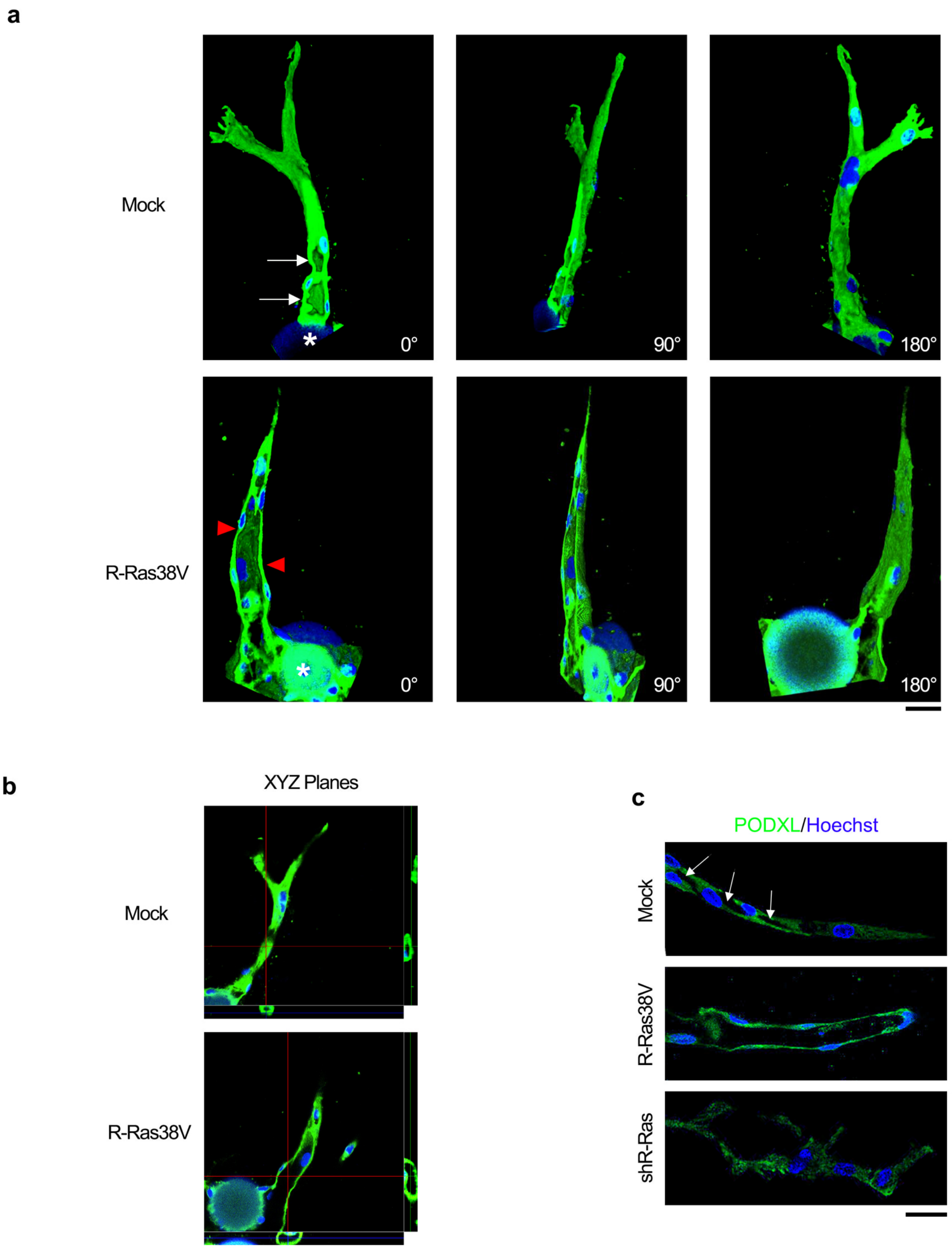

Supplementary Figure 1. R-Ras promotes endothelial lumenogenesis

ECs were transduced with R-Ras38V or control lentivirus (mock), coated onto microbeads, embedded in fibrin gel, and cultured for 5 days. The fibrin gel culture was fixed and stained with UEA lectin (green). **a**, Confocal z-stacks were captured and 3-D images were reconstituted. The Supplementary Videos have rotating images to show the lumen (view of interior) and vessel wall (view from outside). For creating these videos, Z-sections were taken up to the half way of the sprout's diameter in order to view the interior of cylindrical structure of the EC sprout. Snapshots of rotating sprouts were taken at three different angles and presented here. The 0° angle shows the lumen and the 180° angle shows the endothelial wall from the back view. The R-Ras38V-transduced ECs produced a large lumen encased by thin endothelial wall with flattened nuclei (red arrowheads). Mock-transduced ECs produced smaller hallow compartments (arrows). *microbeads. **b**, The XY plane (large center panel), XZ plane (small bottom panel), and YZ plane (small right panel) of confocal images are presented to view cross-sections of the EC sprouts. **c**, PODXL immuno-staining of endothelial sprouts. The middle panel shows a lumen structure with thin endothelial wall with flattened nuclei formed by R-Ras38V-expressing ECs. Arrows, mock control ECs in the process of forming lumen. Scale bars, 25μm.

a

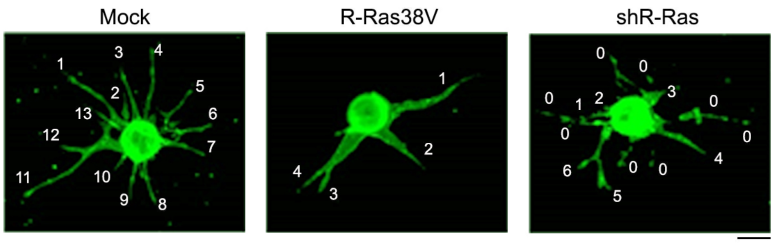

b

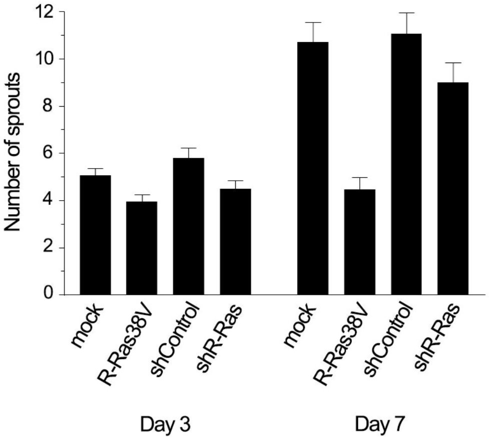

Supplementary Figure 2. R-Ras reduces the number of endothelial sprouting and branching while promoting lumenogenesis.

ECs were transduced with R-Ras38V or mock control lentivirus or R-Ras silenced by shRNA (shR-Ras), coated onto microbeads, and embedded in fibrin gel 3-D culture with a feeder layer of pericytes on the top. The number of sprout formation was determined by counting the sprout ends after in-gel staining with fluorescently labeled lectin (green). **a**, examples of counting. The R-Ras silenced culture produced many ECs and sprouts, which were dissociated or independently migrating out from the stem of the sprouts. These were marked as 0 and not included in the count. **b**, Sprout count per bead on Day 3 and Day 7 of the culture. Scale bar, 50μm.

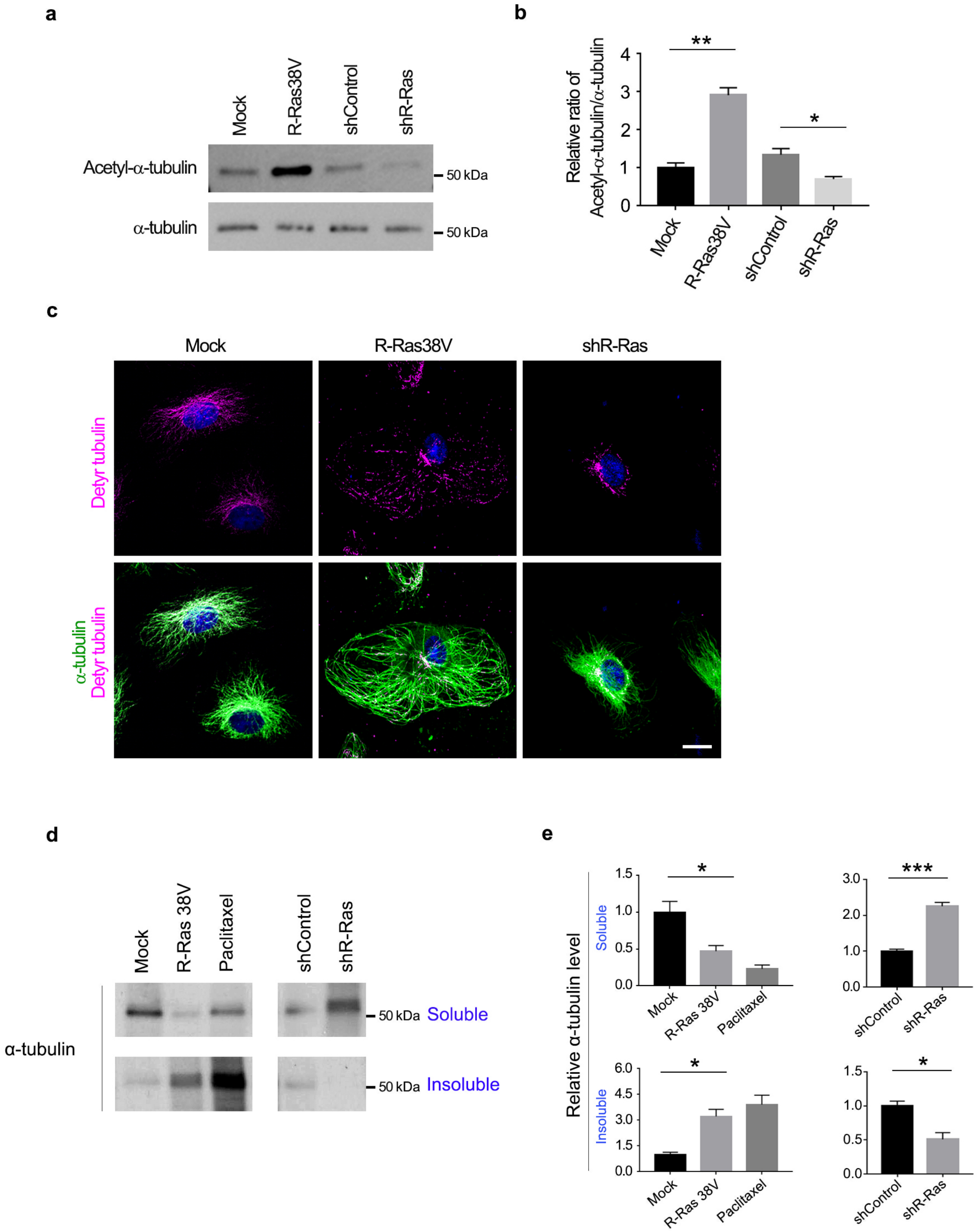

Supplementary Figure 3. R-Ras regulates stability of endothelial microtubules.
a, b, Western blot analysis for total and acetylated α-tubulin (Acetyl-α-tubulin). **c**, Immunofluorescence of total and detyrosinated (Detyr) α-tubulin. The detyrosinated α-tubulin distribution extends to the cell membrane in R-Ras38V-transduced ECs. Scale bar, 25μm. **d**, Detergent fractionation experiment. The soluble (cytosolic) and insoluble (cytoskeletal) fractions were prepared by 0.5% Triton X-100 cell lysis followed by centrifugation. The relative amount of α-tubulin in each fraction was determined by western blot. Paclitaxel-treated EC culture (5 ng/ml) was used as a control for microtubule stabilization. **e,** Quantification of α-tubulin in soluble and insoluble fractions. The images are representative of at least three independent experiments, and the graphs show the quantification of combined results. Data are presented as mean ± s.e.m. $*p < 0.05$, $**P < 0.01$ and $***p < 0.001$.

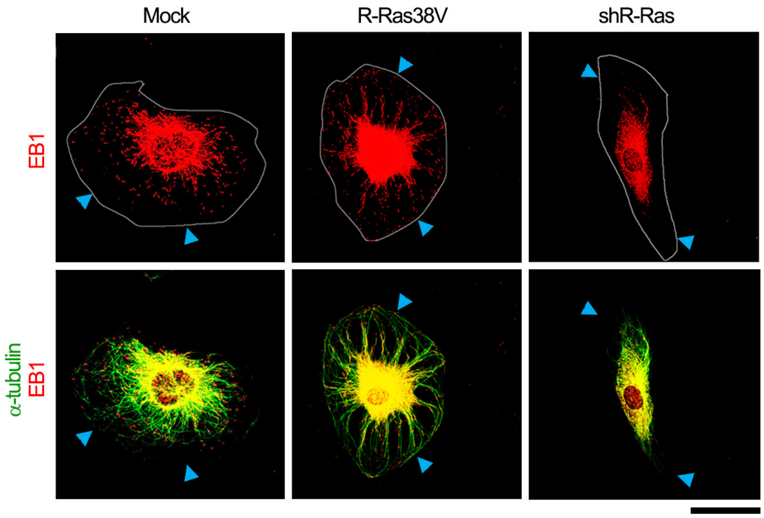

Supplementary Figure 4. R-Ras facilitates microtubules to reach membrane periphery. Immunofluorescence of α-tubulin and microtubule end-binding protein (EB1) to indicate the (+) ends and length of microtubules in monolayer cultures of ECs. Thin gray lines and arrowheads indicate the outlines of cell membrane. Scale bar, 50μm.

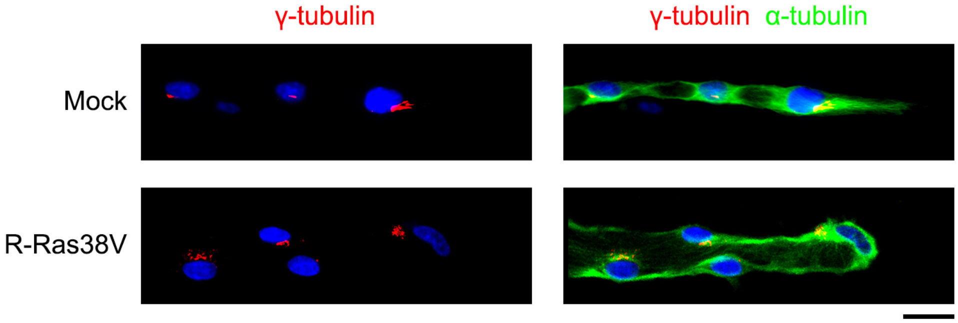

Supplementary Figure 5. Cell polarity in lumenized endothelial sprouts.
The control or R-Ras38V-expressing endothelial sprouts in 3-D culture were stained for γ-tubulin immunofluorescence (red) in order to mark the microtubule organizing center (MTOC). MTOCs are positioned at the apical side of nuclei in the sprouts with lumen, indicating the polarization of ECs. α-tubulin is shown in green. Scale bar, 25 μm

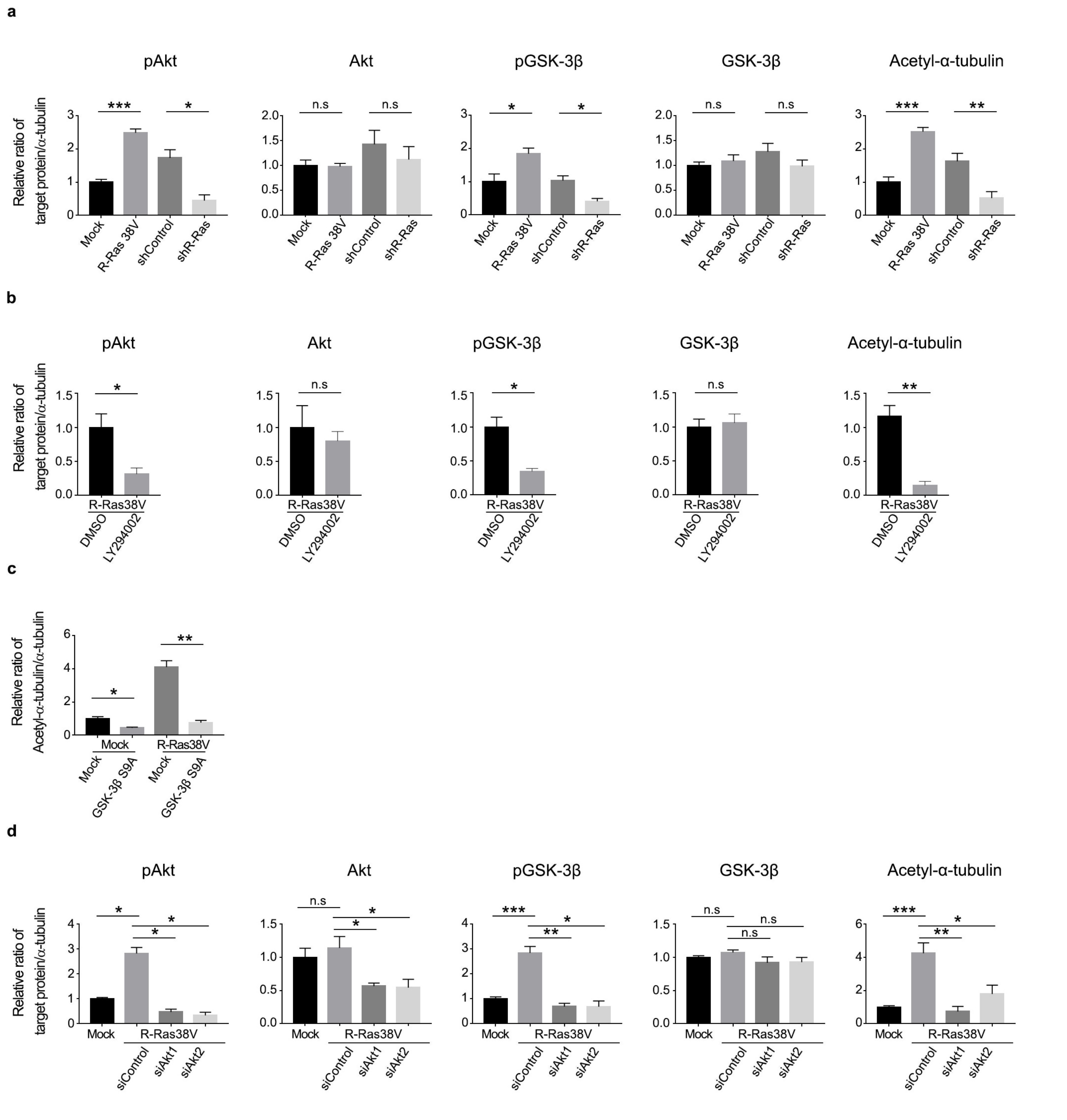

Supplementary Figure 6. **R-Ras-dependent Akt signaling and microtubule modification.**
Summarized bar graphs of Western blots (shown in Figure 2) for pAkt, total Akt, pGSK-3β, total GSK-3β and Acetyl-α-tubulin, presented as ratio of target protein over total α-tubulin. The graphs show combined results from at least three independent experiments. Values are means±s.e.m. *$p < 0.05$, **$P < 0.01$ and ***$p < 0.001$.

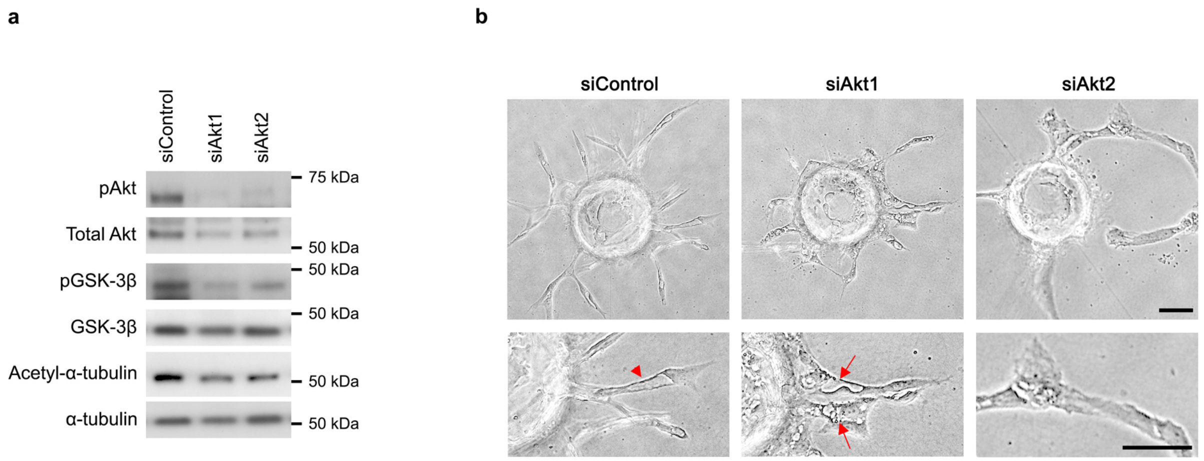

Supplementary Figure 7. Akt isoform knockdown disrupts lumenogenesis of endothelial sprouts
a, siRNA of either Akt isoform (siAkt1 or siAkt2) or control siRNA (siControl) was transfected into parental HUVECs, and Akt and GSK-3β phosphorylation as well as tubulin acetylation were analyzed in these cells 48 hours later by western blot. **b**, The siRNA transfected cells were cultured in 3-D fibrin gel for 5 days to examine the effect of Akt knockdown on lumenogenesis. Arrowhead, lumen formation in the control culture. Arrows, disrupted lumen formation and vacuole accumulations. Scale bars, 25µm.

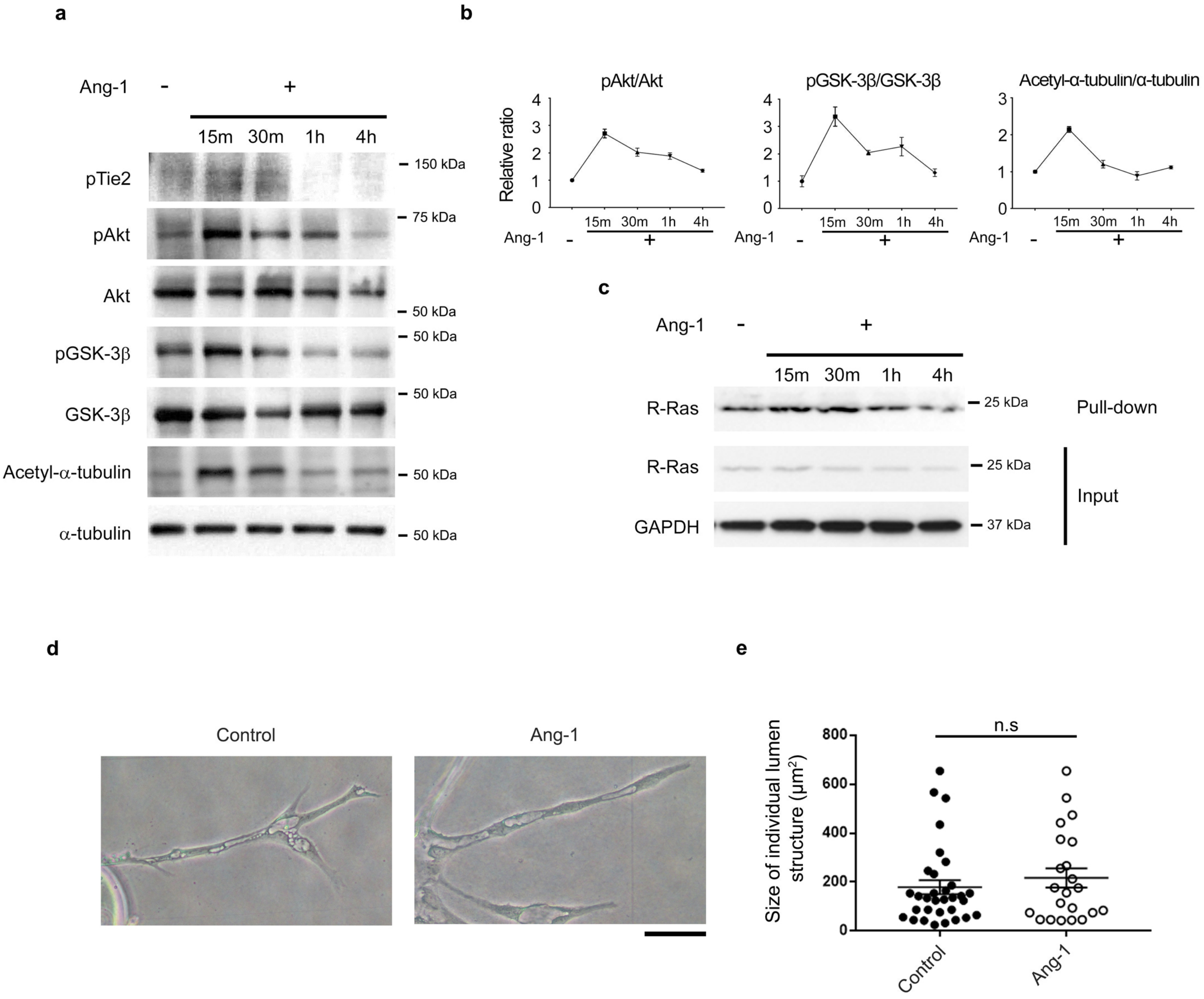

Supplementary Figure 8. The effect of Ang-1 on R-Ras, Akt, and endothelial lumen formation.
ECs were cultured in low-serum media (2% horse serum without growth factor supplement) for overnight and stimulated with (+) or without (-) 500 ng/mL Ang-1 at indicated time points. **a**, Akt (Ser473) and GSK-3β (Ser9) phosphorylation and α-tubulin acetylation were analyzed by Western blot. **b**, Levels of phosphorylated Akt, GSK-3β and acetylated α-tubulin were quantitated by densitometry and normalized to the corresponding total protein levels. The graphs show the combined results of at least three independent experiments. **c,** R-Ras activity was analyzed by pull-down assay at various time points. Input = total cell lysate. **d**, ECs were cultured in 3-D fibrin gel in growth medium with or without additional Ang-1 for 5 days. Scale bars, 25μm. **e**, Quantification of lumen size. The hallow structures of > 5 μm length were considered as developing lumens. The area size of the individual lumen structure was determined by morphometry analysis of bright-field pictures and presented as each plot on the graph. Sprouts grown from 10 EC-coated beads in two different culture wells were analyzed for each group. Small vacuoles of < 5 μm in length were not included in the analysis. Spouts without lumen were not examined. N.S., no significant difference.

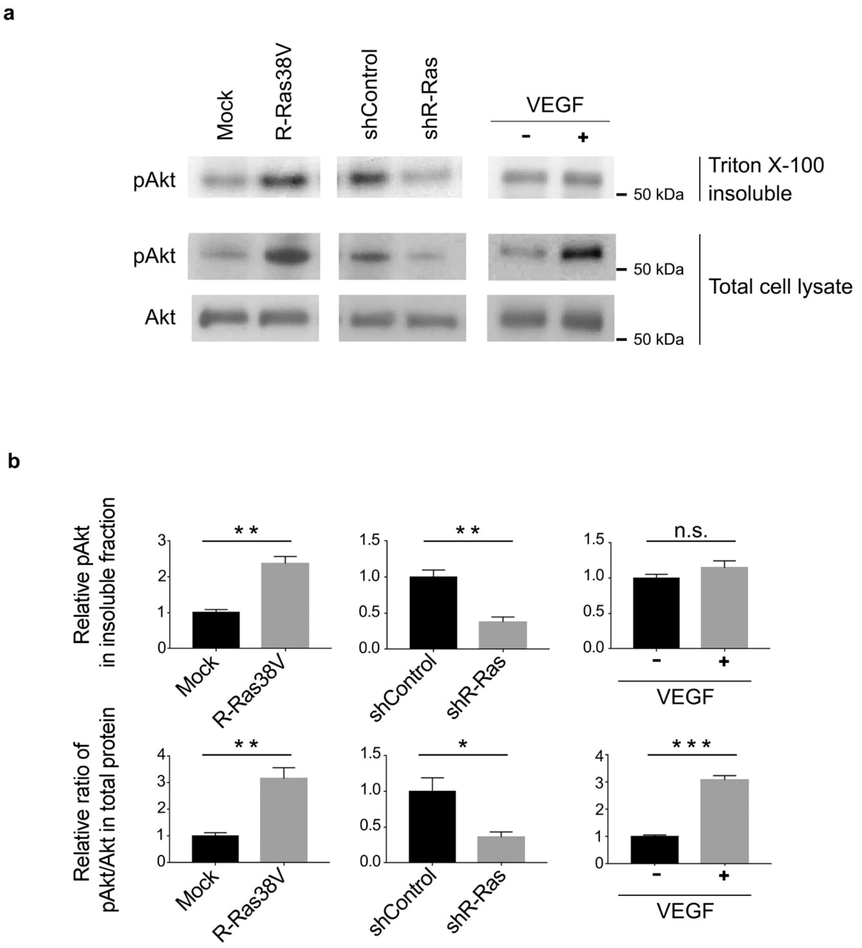

Supplementary Figure 9. Accumulation of activated Akt in detergent-insoluble fraction.
a, ECs were transduced with R-Ras38V (left panel), silenced for endogenous R-Ras by shRNA (middle panel), or stimulated with (+) or without (-) 50 ng/ml VEGF-A for 30min (right panel). The detergent-insoluble cytoskeletal fraction was prepared by 0.5% Triton X-100 cell lysis followed by centrifugation. The relative amount of Ser 473-phosphorylated Akt (pAkt) was determined by western blot. Western blot of total Akt and pAkt in the total cell lysate shows activation of Akt in the cells. **b,** Quantitation of the level of pAkt in insoluble fraction and the ratio of pAkt/Akt in total cell lysates. The western blots are representative of three independent experiments, and the graphs show the quantification of combined results. Data are presented as mean ± s.e.m. $*p < 0.05$, $**P < 0.01$ and $***p < 0.001$.

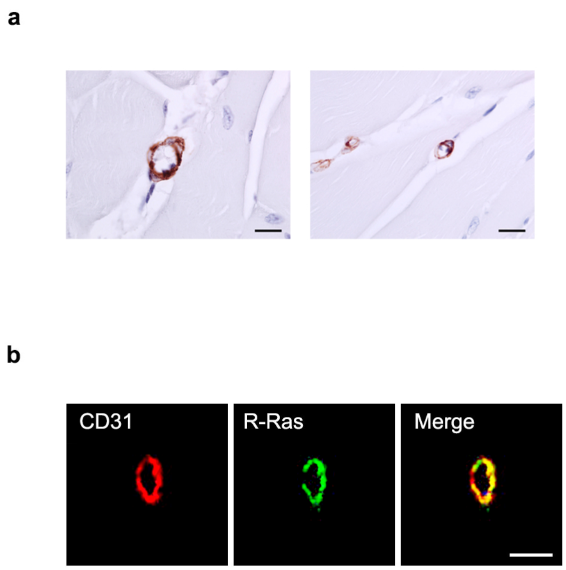

Supplementary Figure 10 R-Ras expression in intramuscular capillaries and microvessels
a, Immunohistochemistry staining of R-Ras in GC muscles of wild type mice showing an intramuscular capillary vessel (left) and microvessels (right). Scale bars, 10 µm. **b**, Immunofluorescence of R-Ras. The wild-type GC muscles were stained for CD31 (red) and R-Ras (green) and analyzed by confocal microscopy. Scale bar, 10 µm.

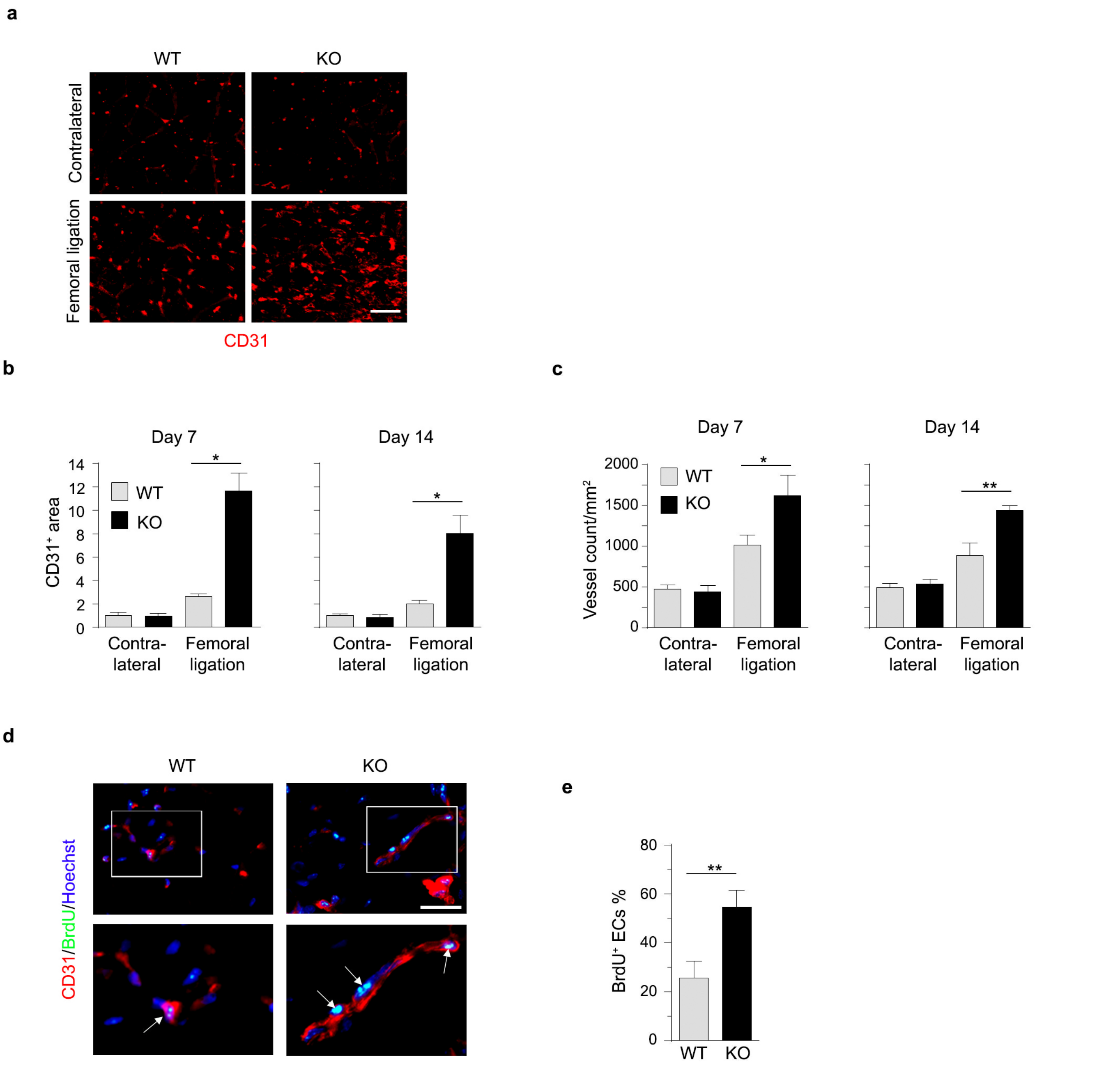

Supplementary Figure 11. R-Ras deficiency exaggerates vessel sprouting in response to acute hindlimb ischemia
a, CD31 staining of GC muscle cross sections at Day 14 post ischemia induction by femoral artery ligation. **b**, Quantification of $CD31^{+}$ area at Day 7 and 14 presented as relative values in comparison to the contralateral wild-type GC muscles. *$p < 0.01$, $n = 5$. **c**, Vessel density. *$p = 0.05$, **$p = 0.01$, $n = 5$. **d**, Immunofluorescence staining of GC muscle cross sections showing ECs that are positive for both BrdU and Hoechst (arrows)**. e**, Percentage of mitotic ($BrdU^{+}$) ECs in GC muscle vasculature at Day 14. Values are means±s.e.m. **$p < 0.01$, $n = 3$. Scale bars, 50 µm.

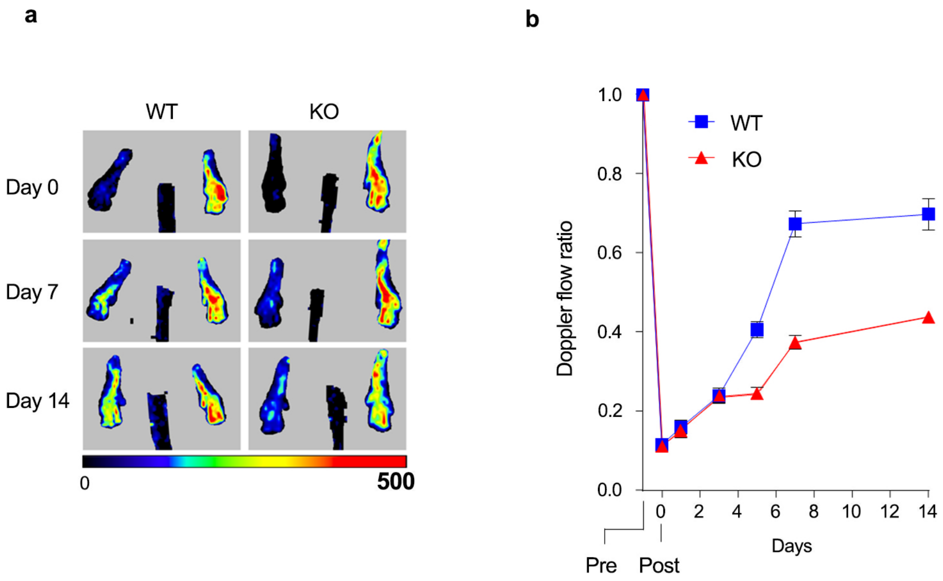

Supplementary Figure 12. Laser Doppler blood flow analysis

The blood flow ratio of ischemic (left) to contralateral (right) feet was determined pre- and post-femoral artery ligation. n = 17. Error bar, s.e.m.

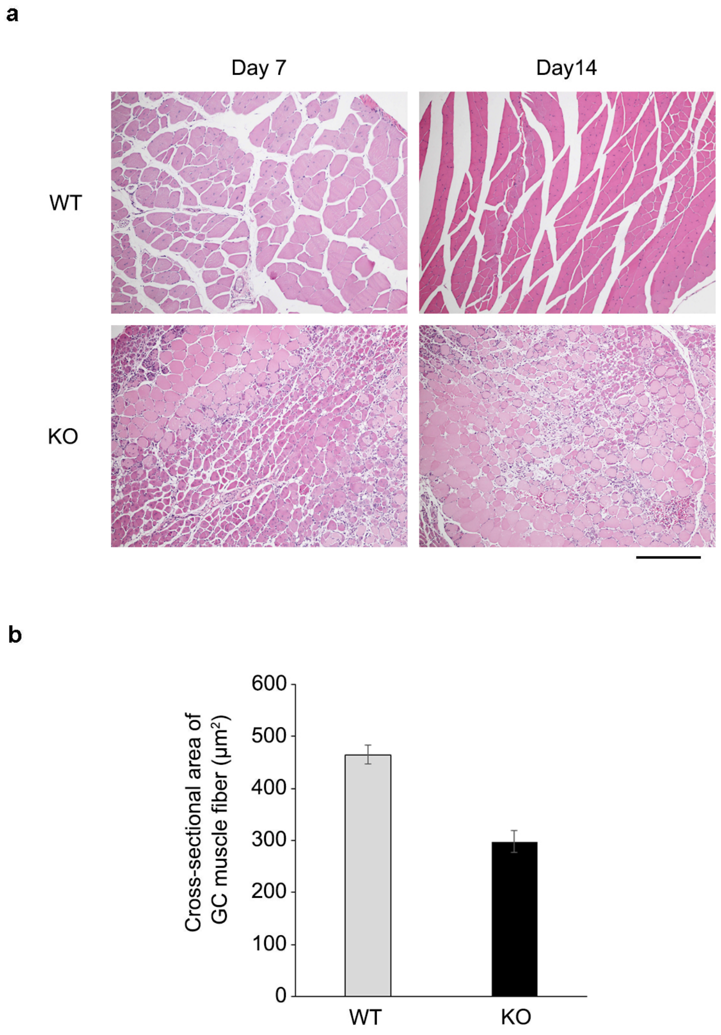

Supplementary Figure 13. Extensive muscle necrosis and lack of recovery associated with defective endothelial lumenogenesis

a, H&E staining of GC muscle sections from wild-type and R-Ras KO mice at 7 and 14 days after ischemia induction, viewed in low magnification. Scale bar, 200 µm. **b**, Comparison of cross-sectional area of muscle fibers in H&E stained GC muscle from WT and KO mice at 14 days after ischemia induction. Muscle fibers in non-necrotic area were analyzed. n=5. Values are means±s.e.m. $P < 0.01$.

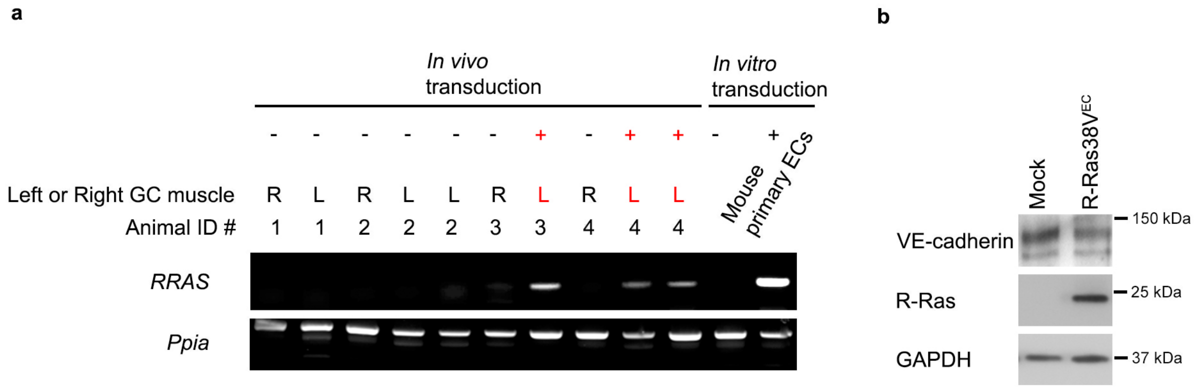

Supplementary Figure 14. Validation of in vivo R-Ras transduction

The left femoral artery was ligated in R-Ras KO mice. The lentivirus carrying pLenti6/*Cdh5*-R-Ras38V expression vector was injected into the left (ischemic) GC muscle 3 days later. GC muscles were resected from both left and right legs at 14 days after femoral artery ligation. **a**, Validation by RT-PCR analysis. One or two fascicles were isolated from each GC muscle for RNA extraction. The muscle RNA was analyzed by RT-PCR for human *RRAS* transgene expression. The mouse *Ppia* expression was used as a standardizing control. An example with four R-Ras KO mice is shown here (Animal ID #1-4). The left GC muscles that received lentivirus injection are indicated in red (3L, 4L). Two pieces of muscle fascicles were examined for the left GC muscles of Animal #2 and 4. Primary culture of R-Ras KO mouse brain microvascular ECs was infected in vitro with the pLenti6/*Cdh5*-R-Ras38V lentivirus as a positive control of transduction (rightmost lane). **b**, Validation by western blot analysis. ECs were isolated by anti-CD31 magnetic bead sorting from the GC muscles that received pLenti6/*Cdh5*-R-Ras38V lentivirus (R-Ras38V^{EC}) or control virus injection (mock). The lysate of these cells were analyzed for R-Ras expression by western blot.

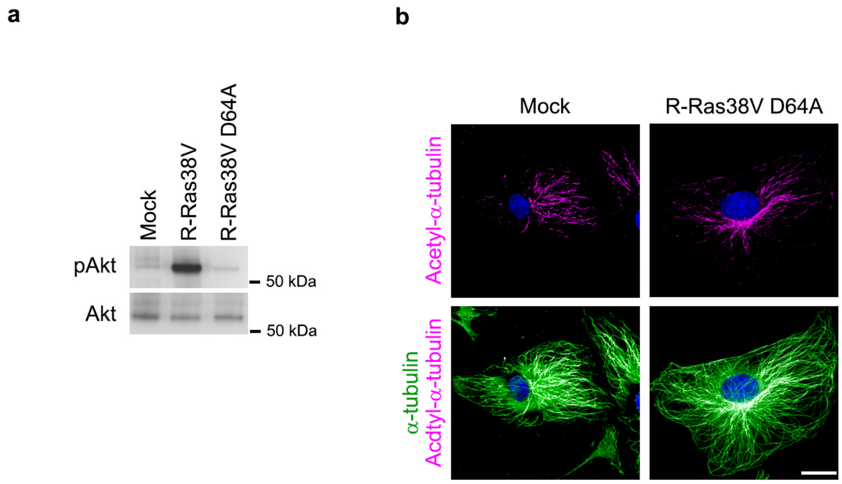

Supplementary Figure 15. R-Ras38V D64A mutant does not activate Akt or stabilize microtubules
ECs were transduced with R-Ras38V or R-Ras38V D64A and analyzed 48 hours later. **a**, Western blot for total and phospho-Akt (Ser473). **b**, Immunostaining of total (green) and acetylated (magenta) α-tubulin. Scale bar, 25 μm

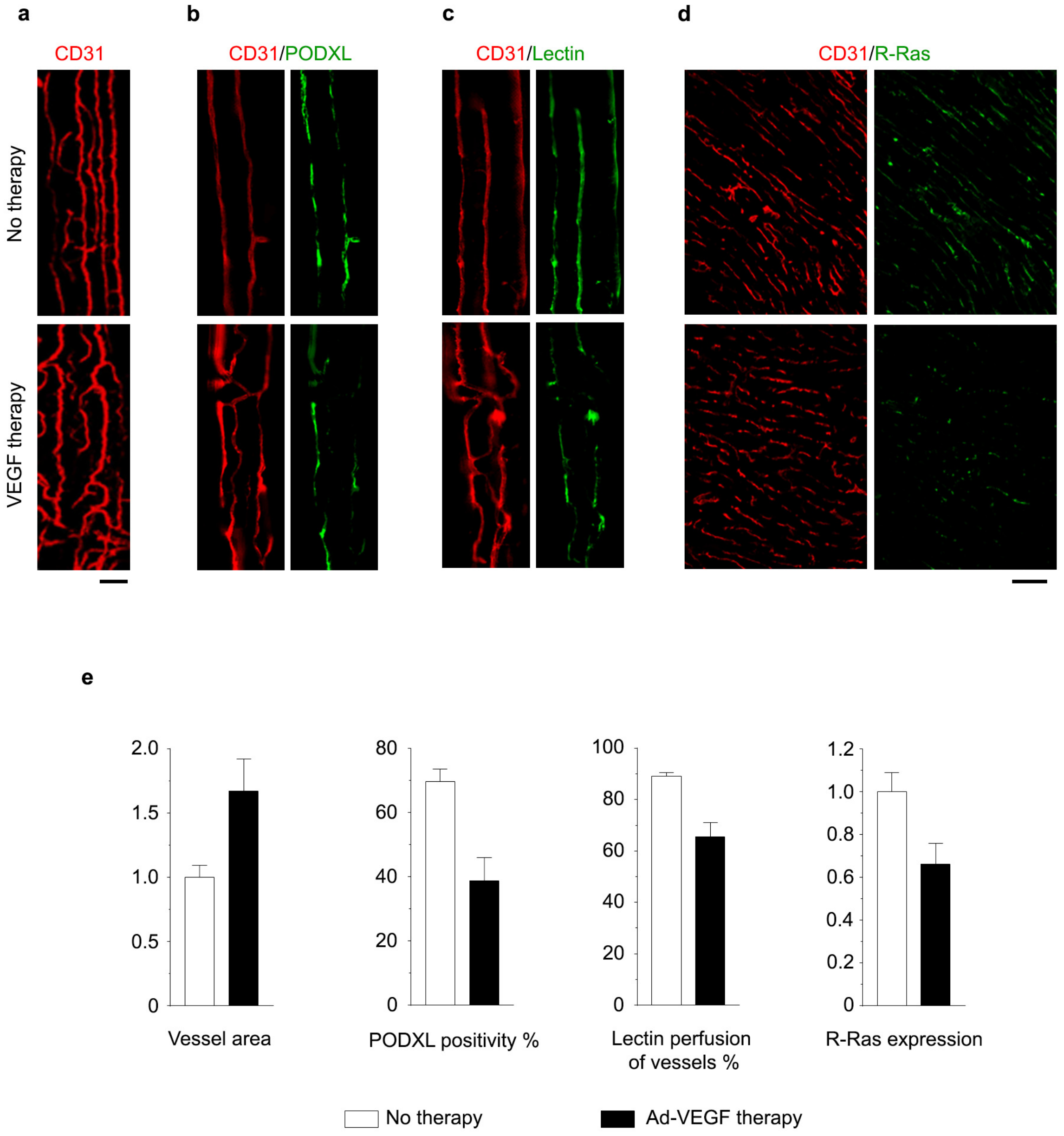

Supplementary Figure 16. Reduced R-Ras expression and insufficient lumenogenesis during angiogenesis induced by Ad-VEGF gene therapy

Immediately after femoral ligation, adenovirus carrying the mouse VEGF-A164 was injected in a small volume into three locations in the GC muscles of wild type C57BL6 mice using Hamilton syringe (1×10^8 pfu/10 µl × 3 injections). The GC muscles were harvested for immunofluorescence analyses 11 days later. **a**, CD31, confocal 3D reconstruction. **b**, **c**, Immunostaining in 50 µm-thick sections. **d**, 10 µm-thick sections. **e**, Quantitative analyses of a-d. Vessels area = $CD31^+$ area in the muscle section presented as relative values with the control group as 1; PODXL positivity % = $PODXL^+CD31^+$ area/$CD31^+$ area × 100; Lectin perfusion % = $lectin^+CD31^+$ area/$CD31^+$ area × 100; R-Ras expression = R-Ras staining integrated intensity in $CD31^+$ area/$CD31^+$ area, presented as relative values; $p < 0.01$ for all comparisons. Scale bars, 15 µm (a-c), 50 µm (d)

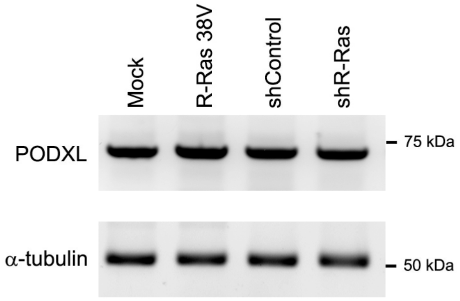

Supplementary Figure 17. The effect of R-Ras on PODXL expression
ECs were transduced with R-Ras38V or silenced for endogenous R-Ras by shRNA (shR-Ras). PODXL protein was analyzed by western blot.

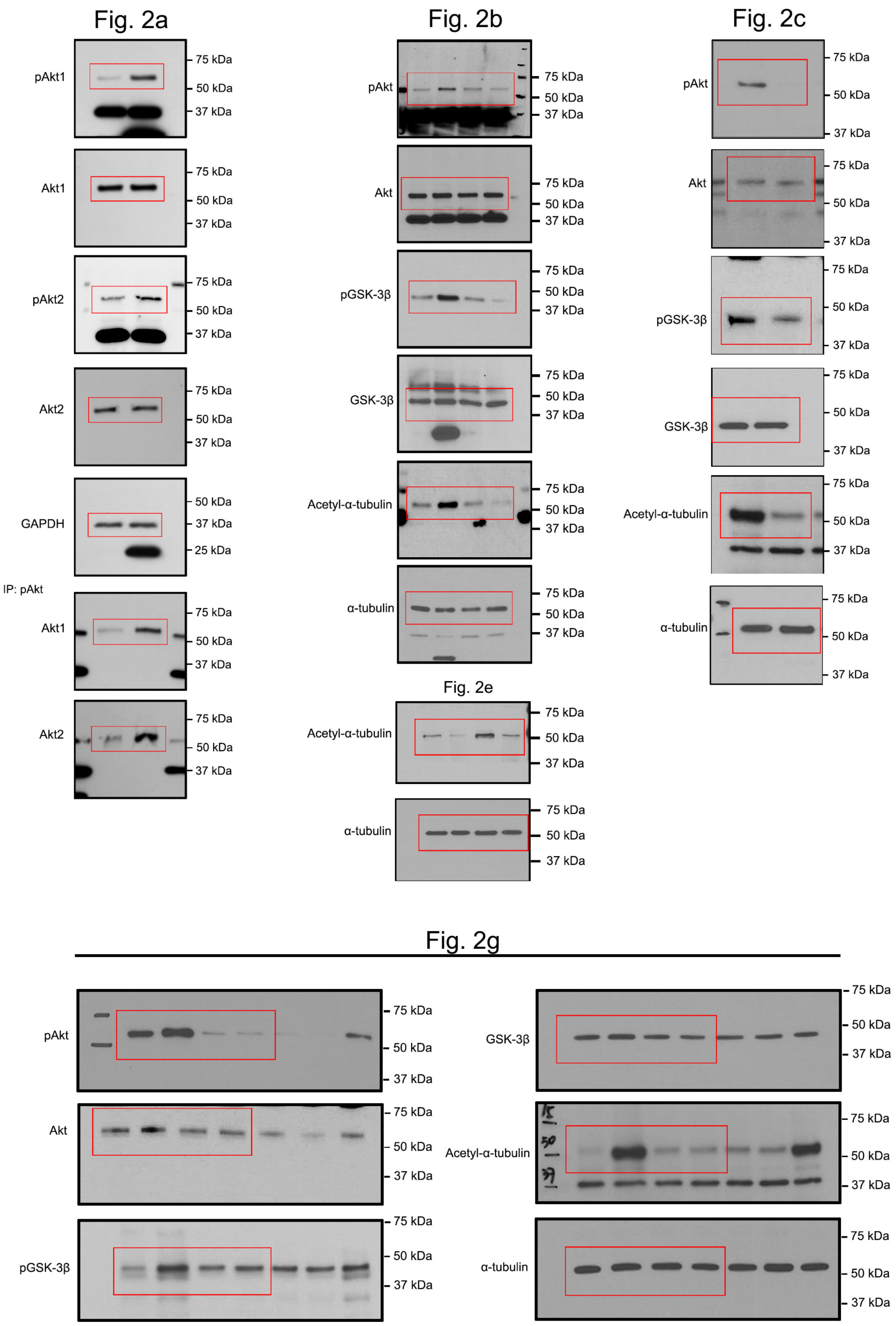

Supplementary Figure 18. Uncropped images of the original scans of Western blots. Uncropped, full-size scans of immunoblots shown in Figure 2.

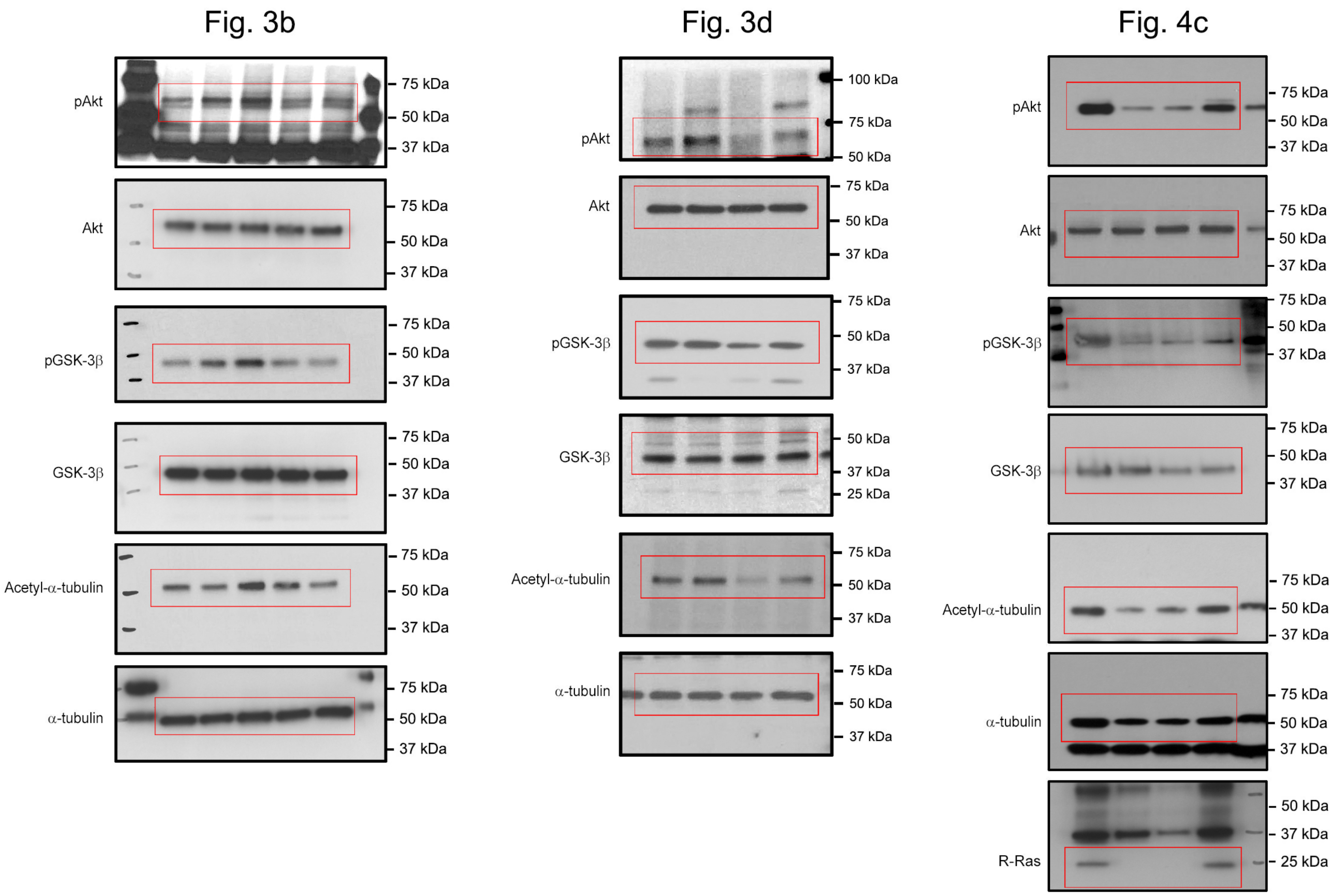

Supplementary Figure 19. Uncropped images of the original scans of Western blots.
Uncropped, full-size scans of immunoblots shown in Figure 3 and Figure 4.
