# Supplementary material for: R-Ras-Akt axis induces endothelial lumenogenesis and regulates the patency of regenerating vasculature
Source: Nat Commun. 2017 Nov 23;8:1720. doi: 10.1038/s41467-017-01865-x (PMC5700916; doi:10.1038/s41467-017-01865-x)
Supplement: Supplementary file 3 — Description of Additional Supplementary Files [file 41467_2017_1865_MOESM3_ESM.pdf]

Description of Supplementary Files

File name: Supplementary Movie 1
Description: Confocal 3-D image of control endothelial sprout

File name: Supplementary Movie 2
Description: Confocal 3-D image of R-Ras38V- expressing endothelial sprout
